# Pericardial Angiosarcoma: A Diagnostic Challenge

**DOI:** 10.7759/cureus.15350

**Published:** 2021-05-31

**Authors:** Sriviji Senthil Kumaran, Abuzar A Asif, Habiba Hussain, Tulika Chatterjee

**Affiliations:** 1 Internal Medicine, University of Illinois College of Medicine Peoria, Peoria, USA; 2 Internal Medicine, OSF Saint Francis Medical Center, Peoria, USA

**Keywords:** pericardial angiosarcoma, angiosarcoma, cardiac angiosarcoma, cardiac tumor, pericarditis, recurrent pericarditis

## Abstract

Cardiac angiosarcomas are the most common malignant primary cardiac tumors accounting for 31% of all primary cardiac tumors. However, primary pericardial angiosarcomas are extremely rare and are associated with high mortality.

A 41-year-old male with a past medical history of end-stage renal disease (ESRD) on hemodialysis, follicular thyroid carcinoma, hypertension, and asthma presented with recurrent pericardial effusions. Previously, different imaging modalities had shown small hemodynamically stable pericardial effusions with pericardial thickening. His pericardial effusion was attributed to his ESRD until this presentation. However, during the current admission, chest X-ray showed cardiomegaly with lobulated left heart border. Computed tomography (CT) and transthoracic echocardiogram showed an increased posterior complex pericardial effusion when compared to previous imaging. A pericardial window was created and the space was evacuated. Pericardial fluid cytology reported rare atypical cells, which were indeterminate for malignancy. Histopathology from the pericardial biopsy revealed fibrous tissue with cellular proliferation, consistent with an angiosarcoma. A positron emission tomography revealed findings of angiosarcoma (hypermetabolic pericardial soft tissue nodularity and complex pericardial fluid) limited only to the pericardium. Unfortunately, the angiosarcoma was deemed unresectable, and the patient underwent one cycle of chemotherapy with paclitaxel. The patient’s hospital course was further complicated by myelosuppression from chemotherapy and disseminated intravascular coagulation. Therefore, no further chemotherapy was pursued. A repeat echocardiogram showed constriction of both ventricles with loculated pericardial effusion. The patient eventually transitioned to comfort care and passed away.

Through this case report, we would like to highlight that multiple confounders can be present when considering the etiology of persistent pericardial effusions. We suggest contemplating alternate diagnosis, such as malignancy, and initiate aggressive work-up especially in young individuals with recurrent, unexplained pericarditis.

## Introduction

Acute pericarditis is a common clinical entity diagnosed when two of the four findings are present: chest pain, pericardial friction rub, diffuse concave upward ST segment elevation on electrocardiogram (EKG), and pericardial effusion on echocardiogram [1]. Recurrence after an acute episode occurs in at least 20-50% of the patients [2]. With the advances in medicine, immunization, and treatment of infections such as tuberculosis, the etiologies of acute pericarditis have changed. Malignant etiology of pericardial effusions is becoming increasingly common. Malignant pericarditis is also more commonly recurrent and presents with tamponade features. Diagnosis and management of these effusions, therefore, usually require invasive procedures such as pericardiocentesis or pericardial window creation. Biopsy with histopathological analysis is the gold standard test and aids in obtaining a pathological diagnosis.

## Case presentation

A 41-year-old African American man presented with a past medical history of uncontrolled hypertension, hypertensive nephrosclerosis, and end-stage renal disease (ESRD) on hemodialysis presented with gradually worsening non-radiating, non-exertional left-sided chest pain that was worse with lying flat and improved on sitting up. His other past medical history included a recent diagnosis of indeterminate follicular neoplastic nodule of the thyroid gland, which was identified incidentally during work-up for possible renal transplant. This was treated with resection of the nodule one month ago. He also had a history of recurrent pericarditis, with his first episode occurring approximately one and half years ago and attributed to uremic pericarditis. At that time, he was treated with aggressive hemodialysis and steroids, which led to temporary resolution of symptoms. Despite compliance with dialysis sessions and being consistently lower than his dry weight, he continued to develop repeated episodes of recurrent pericarditis. On examination during this visit, the patient was hemodynamically stable with no pulsus paradoxus. A pericardial friction rub was heard over the left sternal border.

**Figure 1:**
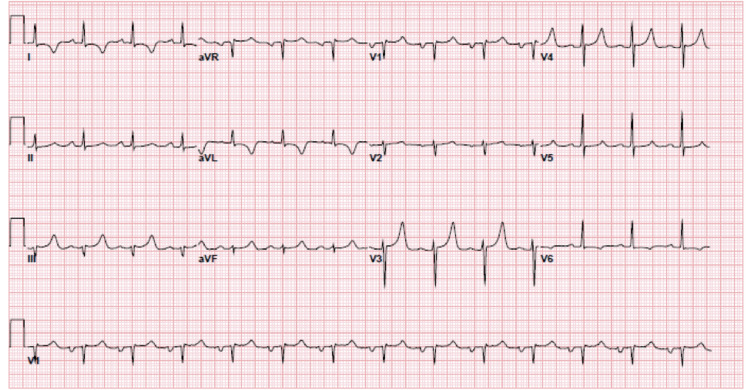
EKG showing normal sinus rhythm with possible left atrial enlargement, with T wave inversions over I, aVL, and V6, and peaked T waves over V3 and V4.

**Figure 2:**
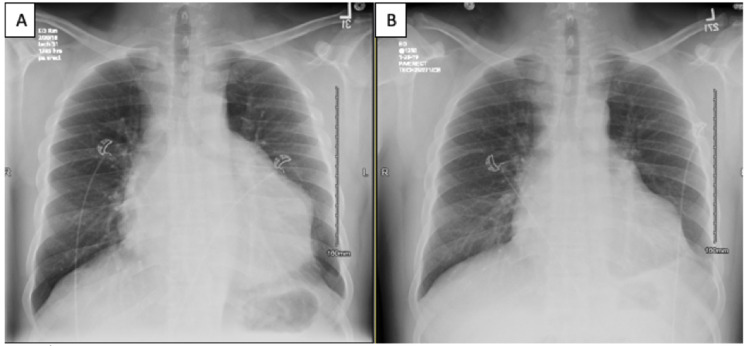
Image A showing an increase in size of cardiomegaly with persistent loculation of the left cardiac margin with worsening pericardial effusion. Persistent small pleural effusion is unchanged compared to imaging performed one month ago (image B).

**Figure 3:**
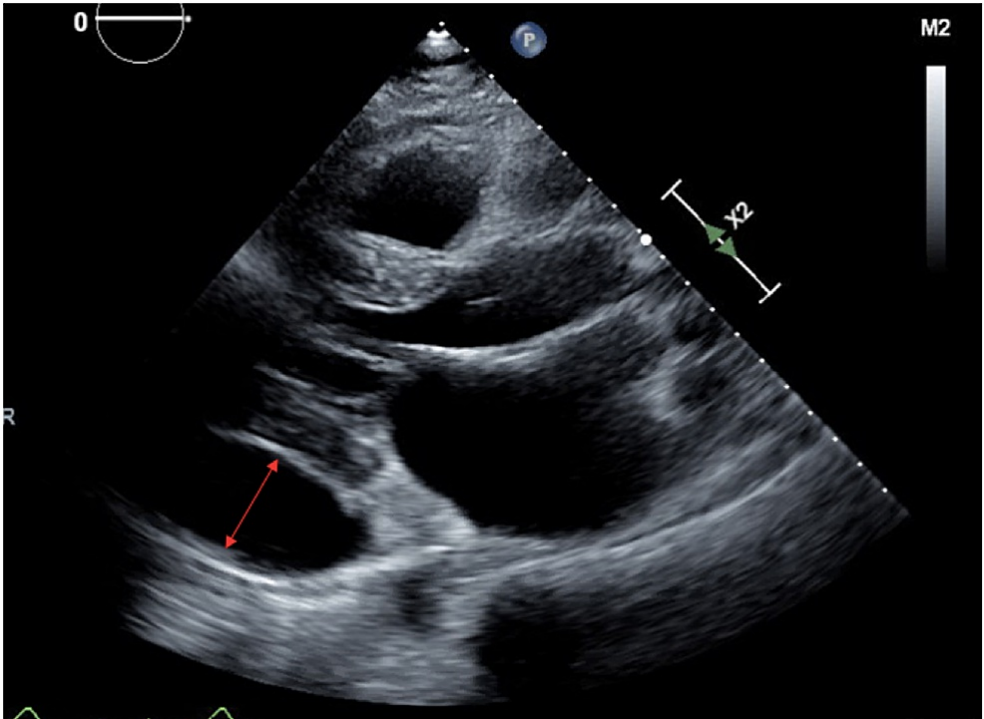
Transthoracic echocardiographic imaging showing moderate-sized posterior pericardial effusion.

**Figure 4:**
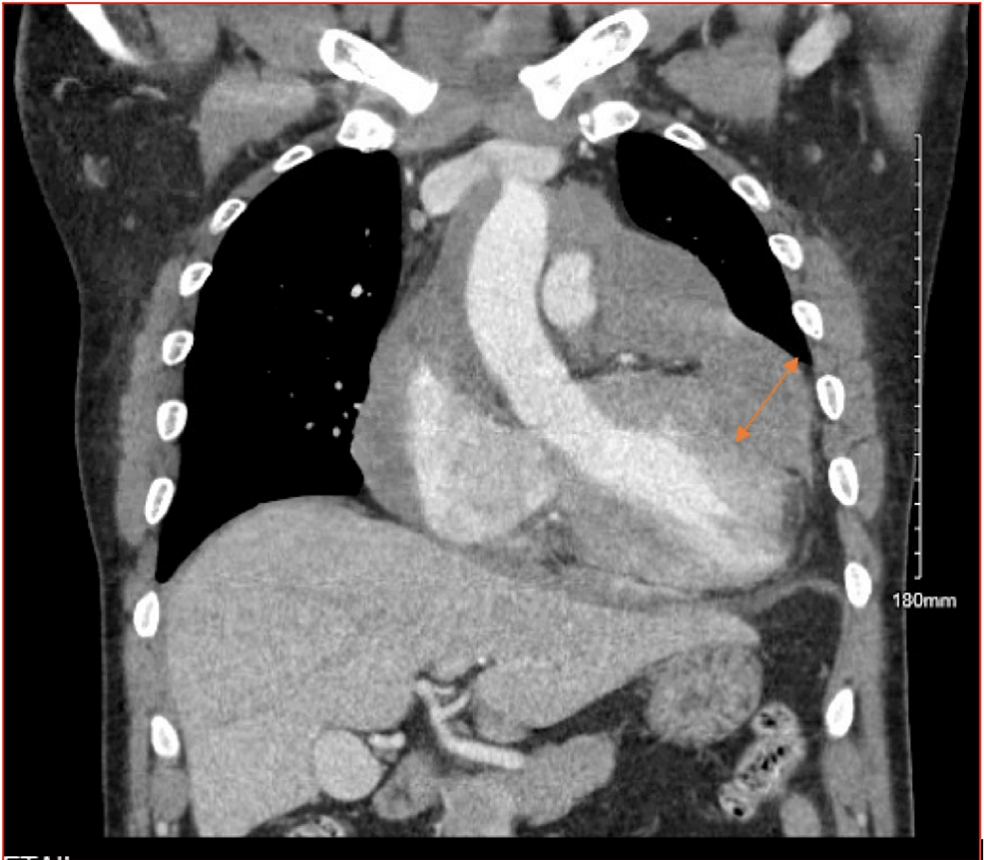
CT of the chest with contrast (coronal view) showing moderate-sized loculated pericardial effusion laterally that appears heterogenous.

**Figure 5:**
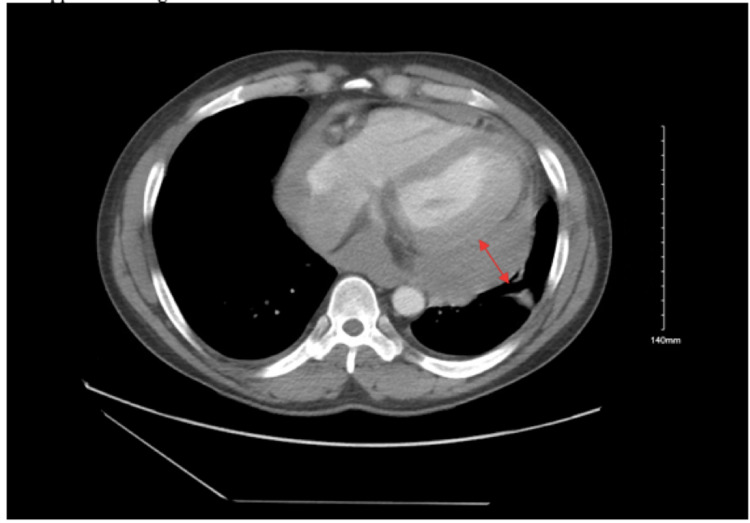
CT of the chest with contrast (axial view) showing posterolateral loculated pericardial effusion, which measures 36 mm.

Initial laboratory work-up was remarkable for mild microcytic anemia, thrombocytopenia, elevated creatinine, blood urea nitrogen (BUN), mildly elevated troponin, and elevated C-reactive protein (CRP) (values listed in Table 1). EKG (Figure 1) showed normal sinus rhythm with possible left atrial enlargement, with T wave inversions over I, aVL, and V6, and peaked T waves over V3 and V4. Chest X-ray (Figure 2) showed an increase in the size of cardiomegaly with persistent loculation of the left cardiac margin with worsening lobulated pericardial effusion from one month ago. Transthoracic echocardiogram (TTE) showed ejection fraction of 60-65% with mild concentric left ventricular hypertrophy and moderate-sized posteriorly located pericardial effusion (Figure 3). Computed tomography (CT) (Figures 4, 5) showed a loculated pericardial effusion measuring 36 mm, with the largest component at the left lateral and posterolateral pericardial space. Pericardial fluid appeared dense and heterogenous (36 HU). There was a mass effect on the left superior and left inferior pulmonary veins. This imaging had shown significant progression when compared to the previous CT performed one month ago.

**Table 1:** Laboratory work-up at initial presentation WBC, white blood cells; BUN, blood urea nitrogen; BNP, B-type natriuretic peptide; ESR, erythrocyte sedimentation rate; CRP, C-reactive protein; ANA, antinuclear antibodies; ANCA, antineutrophil cytoplasmic antibodies; TSH, thyroid-stimulating hormone

Parameter	Value
WBC	5.39 x 10^3^/mcl with normal differential
Hemoglobin	8.3 g/dL (baseline: 9.3 g/dL): microcytic hypochromic with anisopoikilocytosis
Platelet count	131 x 10^3^/mcl (baseline: 220 x 10^3^/mcl)
Sodium	140 mmol/L
Potassium	4.9 mmol/L
Chloride	94 mmol/L
Bicarbonate	31 mmol/L
BUN	64 mg/dL
Creatinine	12.64 mg/dL (on hemodialysis; last dialysis two days ago)
BNP	886 pg/mL
Troponin	0.056 ng/mL to 0.060 ng/mL to 0.101 ng/mL
ESR	18 mm/h
CRP	4.89 mg/dL
ANA	Negative
Rheumatoid factor	<15 IU/mL
ANCA	Negative
TSH	Normal
D-dimer	<0.15 mcg/mL

Differential diagnosis

Elevated cardiac biomarkers are common in ESRD patients. The atypical nature of the pain, benign troponin trend, and lack of EKG changes made acute coronary syndromes less likely. Clinical findings and imaging detailed above ruled out aortic dissection. The lack of jugular venous distension, peripheral edema, normal systolic, and diastolic function ruled out heart failure as a cause for the pericardial effusion. Elevated BNP can be seen in patients with constrictive physiology especially in the setting of chronic kidney disease [3,4]. The negative inflammatory markers and negative autoimmune work-up made autoimmune etiology for the pericardial effusion less likely. Pericardial effusions secondary to ESRD can be uremic pericarditis and dialysis pericarditis [5]. Uremic pericarditis typically occurs prior to and within eight weeks of initiation of hemodialysis due to accumulation of toxic metabolites. Dialysis pericarditis occurs due to an inadequate or missed dialysis treatment [5,6]. Our patient had not missed any dialysis, and all of these sessions had been adequate. Moreover, the presence of >30 HU on CT confirms the presence of proteinaceous fluid/ hemopericardium. ESRD patients are at an increased risk of constrictive pericarditis due to repeated hemopericardium resulting in scar tissue formation. This is in part attributed to platelet dysfunction and altered coagulopathy and fibrin system, which are widely prevalent in these patients [5]. There was also concern for primary or secondary malignant effusion. The patient’s weight loss and recent diagnosis of follicular thyroid neoplasm increased this likelihood. However, a malignant effusion is more common with papillary thyroid carcinoma [7]. Other less likely differentials included an autoinflammatory disease such as IgG4 (immunoglobulin G4) disease and other primary or secondary malignancies of the pericardium.

Management

The patient was started on prednisone 0.5 mg/kg/day following with which his chest pain improved. Left heart catheterization was unremarkable. Hemodynamics on right heart catheterization (Table 2) showed no evidence of constriction. Given the progressive increase in size, posterior location, and loculated and complex appearance with progressive symptoms of hypotension and palpitations, a pericardial window was created. A hemorrhagic effusion of 50 cc with many clots was drained. Extremely adherent clots were found in the superior aspect around the pulmonary vein, left atrial appendage, and left pulmonary artery, which were not resected. A 4 x 2 cm mass found above the phrenic nerve was excised. A pericardial drain was placed. Biopsies of the above were sent for histopathological and cytological analysis. Meanwhile, the patient was continued on a prednisone taper. A chest tube was also placed in the left pleural space.

**Table 2:** Right heart catheterization findings

Parameters	Value
Right atrium pressure	5 mmHg
Right ventricle pressure	48/10 mmHg
Pulmonary artery pressure	40/16 mmHg
Left ventricle pressure	105/21 mmHg
Ejection fraction	60%
Cardiac output: fick	4.95 L/min
Cardiac output: thermal	4.766 L/min

Cytology from the pericardial fluid revealed rare, atypical cells that were indeterminate for malignancy. The histopathology of the mass showed blood and fibrin with clusters of malignant epithelioid cells and minimal amounts of cytoplasm, irregular nuclei, and coarse chromatin. Atypical mitosis and punctate necrosis were also identified with the cell populations, and they formed anastomosing vascular channels in some areas. Immunohistochemistry studies showed the neoplastic cells to exhibit strong reactivity to antibodies against ERG, CD31, but no reactivity against antibodies to TTF-1. Weak reactivity was noted against antibodies to Oscar keratin. Conclusively, taking all of the above into consideration, a pathological diagnosis of angiosarcoma was made.

Follow-up

**Figure 6:**
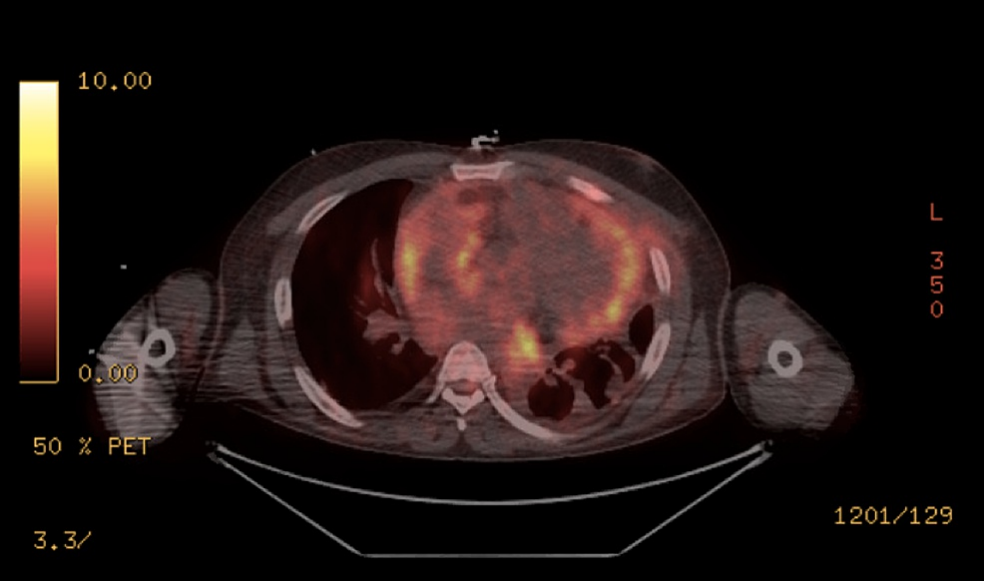
Axial view of PET showing increased FDG uptake in the pericardium PET, positron emission tomography; FDG, fluorodeoxyglucose

Positron emission tomography (PET) (Figure 6) showed that the disease burden was limited to the pericardium, with evidence of some lung nodularity. The disease was deemed unresectable as it was locally advanced and considered stage IV. Overall, given his performance status and comorbid conditions, treatment was initiated with single-agent paclitaxel. He then developed severe pancytopenia and could not tolerate more than a single cycle of chemotherapy. His disease eventually progressed to cause biventricular constriction and eventually developed disseminated intravascular coagulation. Given the aggressiveness of his disease and his poor prognosis, he chose to pursue comfort-focused treatment. He died two months after his diagnosis.

## Discussion

Primary cardiac tumors are rare and account for 0.0027%-0.003% of all tumors during routine autopsy studies [8]. Angiosarcomas are the most common (31%) primary malignant cardiac tumors [9]. These tumors are aggressive in nature but remain asymptomatic initially, resulting in diagnosis at a late stage when the disease progresses to substantial locoregional metastasis. By the time the diagnosis is made, chances of surgical cure are minimal and thus result in poor prognosis. Primary pericardial angiosarcomas are extremely rare and are often misdiagnosed during the early course of the disease, as was the case in our patient. Cardiac angiosarcomas have male preponderance (3:2) with right atrial predilection and occur commonly in those in the age group of 20-50 years [10].

The initial imaging modality used in patients with pericarditis or pericardial effusion is TTE, especially due to its ease of access and convenience. Kupsky et al. reported a single-center experience over a span of 37 years regarding the echocardiographic features of cardiac angiosarcomas [11]. They reported a sensitivity of 75% when performed as the first diagnostic test. They described predominantly non-mobile homogenous masses with broad-based attachment and smooth intracardiac borders most commonly present in the right atrium. These characteristics make identification of pericardial angiosarcomas harder with 2D TTE.

CT identifies broad-based masses that are inhomogeneous without contrast and have heterogenous and centripetal enhancement with contrast [12]. Even with the advent of advanced imaging techniques, this elusive tumor can be easily missed. Shi and Li [13] reported a case of a 62-year-old female with recurrent hemorrhagic effusions who was first identified to have multiple abnormal enhancements limited to different parts of the pericardium with FDG (fluorodeoxyglucose) PET/CT. She was later diagnosed to have primary pericardial angiosarcoma after histopathological confirmation.

Surgical resections are both diagnostic and therapeutic. Pericardial fluid cytology has been consistently negative in all reported cases of cardiac angiosarcomas. Achieving R0 margins (no microscopic cancer cells in the margins of the specimen from the primary site) is the goal for all patients and greatly determines prognosis (median survival of 17 months). This drops to six months with R1 margins (microscopic cancer cells present in the margins) [14-18]. However, the proximity of all lesions to vital cardiac structures makes surgical treatment challenging. No official guidelines are available for the treatment. Chemotherapy in both adjuvant and neoadjuvant settings has been tried with anthracyclines and taxanes. Neither has been compared in a prospective trial, but retrospective studies have shown similar survival benefits [18-20].

## Conclusions

We have been unable to reliably quantify the incidence of pericardial angiosarcomas. This is due to their variable presentation, early misdiagnosis, and rapid progression to death. Echocardiograms are relatively cheap, easily available, and first-line investigations for most pericardial effusions. However, in patients with recurrent pericarditis and/or hemorrhagic effusions receiving appropriate treatment, close follow-up for resolution is indicated. Further imaging with CT or MRI should be considered early. R0 resection is the gold standard treatment, but this will be impossible without early diagnosis. Heterogeneity in the pericardial space almost always indicates debris or blood. Smaller pericardial effusions causing mass effect on the heart need surgical exploration. A multi-disciplinary team of primary care doctors, internists, cardiologists, cardiovascular surgeons, oncologists, and radiologists is very important for the appropriate diagnosis and management of these complex patients.

## References

[1] Imazio M, Demichelis B, Parrini I (2004). Day-hospital treatment of acute pericarditis: a management program for outpatient therapy. J Am Coll Cardiol.

[2] Cacoub P, Marques C (2020). Acute recurrent pericarditis: from pathophysiology towards new treatment strategy. Heart.

[3] Mouyis K, Singer D, Missouris C (2019). Grossly elevated plasma BNP does not exclude the diagnosis of constrictive pericarditis. Oxf Med Case Reports.

[4] Reddy PR, Dieter RS, Das P, Steen LH, Lewis BE, Leya FS (2007). Utility of BNP in differentiating constrictive pericarditis from restrictive cardiomyopathy in patients with renal insufficiency. J Card Fail.

[5] Rehman KA, Betancor J, Xu B (2017). Uremic pericarditis, pericardial effusion, and constrictive pericarditis in end-stage renal disease: Insights and pathophysiology. Clin Cardiol.

[6] Bailey GL, Hampers CL, Hager EB, Merrill JP (1968). Uremic pericarditis. Clinical features and management. Circulation.

[7] Kovacs CS, Nguyen GK, Mullen JC, Crockford PM (1994). Cardiac tamponade as the initial presentation of papillary thyroid carcinoma. Can J Cardiol.

[8] Silverman NA (1980). Primary cardiac tumors. Ann Surg.

[9] Blondeau P (1990). Primary cardiac tumors - French studies of 533 cases. Thorac Cardiovasc Surg.

[10] Kurian KC, Weisshaar D, Parekh H, Berry GJ, Reitz B (2006). Primary cardiac angiosarcoma: case report and review of the literature. Cardiovasc Pathol.

[11] Kupsky DF, Newman DB, Kumar G, Maleszewski JJ, Edwards WD, Klarich KW (2016). Echocardiographic features of cardiac angiosarcomas: the Mayo Clinic experience (1976-2013). Echocardiography.

[12] Chen Y, Li Y, Zhang N (2020). Clinical and imaging features of primary cardiac angiosarcoma. Diagnostics (Basel).

[13] Shi X, Li F (2017). Primary pericardial angiosarcoma shown on FDG PET/CT. Clin Nucl Med.

[14] Lau C, Leonard JR, Schwann AN (2019). A 20-year experience with resection of primary cardiac tumors and metastatic tumors of the heart. Ann Thorac Surg.

[15] Truong PT, Jones SO, Martens B (2009). Treatment and outcomes in adult patients with primary cardiac sarcoma: the British Columbia Cancer Agency experience. Ann Surg Oncol.

[16] Simpson L, Kumar SK, Okuno SH, Schaff HV, Porrata LF, Buckner JC, Moynihan TJ (2008). Malignant primary cardiac tumors: review of a single institution experience. Cancer.

[17] Mayer F, Aebert H, Rudert M (2007). Primary malignant sarcomas of the heart and great vessels in adult patients--a single-center experience. Oncologist.

[18] Yadav U, Mangla A (2020). Primary pericardial angiosarcoma: case report and review of treatment options. Ecancermedicalscience.

[19] Young RJ, Brown NJ, Reed MW, Hughes D, Woll PJ (2010). Angiosarcoma. Lancet Oncol.

[20] Sleijfer S, Seynaeve C, Verweij J (2005). Using single-agent therapy in adult patients with advanced soft tissue sarcoma can still be considered standard care. Oncologist.

